# Effect of Sepiolite on the Field-Dependent Normal Force of Magnetorheological Grease

**DOI:** 10.3390/ma16165627

**Published:** 2023-08-15

**Authors:** Mengwei Du, Huixing Wang, Xudan Ye, Kun Qian, Jiong Wang

**Affiliations:** School of Mechanical Engineering, Nanjing University of Science & Technology, Nanjing 210094, Chinaymxd_li@163.com (X.Y.);

**Keywords:** sepiolite, magnetorheological grease, steady-state shear, oscillating shear, normal force

## Abstract

In order to investigate the influence of sepiolite minerals on the normal force of magnetorheological grease (MRG), a mixed sample (ALCH) on the basis of preparing an aluminum–lithium-based magnetorheological grease (base sample ALC), containing sepiolite was further prepared. The field-dependent normal force of the two samples was tested using a rotational rheometer, considering conditions such as magnetic field, time, strain amplitude, frequency, and temperature, and the results were compared. The results indicate that sepiolite limits the field dependent normal force of the magnetorheological grease under steady state shear, and is unaffected by magnetic field, time, temperature, and shear rate. Sepiolite has minimal impact on the transient response of the magnetorheological grease. Under oscillatory shear, the magnetic field is an important factor influencing the field-dependent normal force response of the sepiolite-magnetorheological grease (ALCH). At low magnetic fields, the field-dependent normal force of the sepiolite-containing sample (ALCH) is greater than that of the base sample (ALC), while this relationship is reversed at high magnetic fields, unaffected by other factors. Under long-term shear conditions, both samples exhibit good shear stability, as well as consistency at different frequencies and strain amplitudes. However, an increase in shear rate reduces the normal force, and temperature also affects the field-dependent normal force. The patterns of variation in steady-state and oscillatory shear modes are not entirely the same, but both exhibit a characteristic decrease with increasing temperature under high magnetic field intensities. Sepiolite can reduce the temperature sensitivity of the normal force of the magnetorheological grease. In conclusion, the introduction of sepiolite is beneficial for the application of magnetorheology in high-precision devices.

## 1. Introduction

Magnetorheological grease is a type of magnetorheological smart material that possesses magnetorheological effects [1,2,3,4]. Magnetorheological grease materials have advantages such as good resistance to settling, leakage prevention, strong magnetorheological effects, and ease of preparation, as their viscosity is based on a combination of liquid magnetorheological fluid and solid magnetorheological elastomers. Therefore, they have found wide application in devices such as dampers [5], buffers [6], clutches [7], and brakes [8], and have promising prospects for further use. However, magnetorheological greases still have some drawbacks, such as high zero-field viscosity and susceptibility to shear-thinning.

Researchers have focused on aspects such as magnetic particle size, base oil viscosity, and additives to study magnetorheological material properties. Poddar et al. [9] investigated the influence of particle size on the properties of magnetorheological materials and found that nanoscale iron particles can produce better suspension and more robust particle chains under the action of a magnetic field compared to microscale iron particles, but nanoscale magnetorheological fluids exhibit lower yield stress. Joanes Berasategi et al. [10] found that ferromagnetic nanoparticles have better magnetic properties than carbonyl iron particles, and the combination of the two shows higher yield stress in magnetorheological fluids. Wang et al. [11] investigated the relationship between the viscosity of base oils with different kinematic viscosities and the zero-field viscosity of lithium-based composite magnetorheological greases, and found that higher kinematic viscosity of the base oil leads to higher zero-field viscosity and improved sedimentation stability. In recent years, the use of clay minerals as additives [12,13,14] has been widely applied in magnetorheological materials, and sepiolite has gradually attracted the attention of scientists. Maurya et al. [15] introduced goethite as an additive to water-based magnetorheological suspensions, resulting in the formation of a clay gel-like structure. This greatly improved the sedimentation stability of the magnetorheological suspension. Chae et al. [16] added palygorskite to magnetorheological grease and observed by electron microscopy that this clay mineral filled the gaps between the magnetic particles, improving the sedimentation and dispersion stability of the carbonyl iron powder. Dong et al. [17,18] prepared iron ore/sepiolite nanocomposite particles and incorporated them into carbonyl iron-based magnetorheological fluids, improving the dispersion and magnetorheological performance of the fluid. Milde R et al. [19] produced a settling stable magnetorheological polishing slurry using sepiolite and Al_2_O_3_ and showed that the addition of sepiolite significantly improved the settling stability of the abrasive solution, reducing the rate of settling to a quarter of the original rate, and that the system was able to maintain long-term stability. However, most research efforts have focused on the shear yield strength and sedimentation stability of magnetorheological materials, with limited investigation into the influence of clay minerals on the normal force in magnetorheological systems, and the study of sepiolite on the normal force remains particularly poor.

It is well known that the normal force is the force perpendicular to the shear stress and is the result of the normal deformation force caused by the magnetorheological effect. It is the basis for the fabrication of mechanical structures and devices with intelligence, adaptability, and controllability [20,21]. The De Vicente, See, and Laun et al. [22,23,24] teams studied the normal force of magnetorheological fluids and magnetorheological elastomers, respectively, and all found that the magnetic field is the main factor influencing the normal force; Guo [25,26] studied the effect of the volume fraction of iron, the magnetic field, the shear rate, and the temperature and other factors on the normal force of magnetorheological fluids, and with Lopez-Lopez [27], reported three different regions of steady state shear behavior. However, the current domestic and international studies on normal force mainly focus on magnetorheological fluids and magnetorheological elastomers, and there are few studies on the normal force in magnetorheological lubricants; however, the fact that magnetorheological greases exhibit superior settling and stability properties has led to their wide application in device design. With the gradual application of magnetorheological greases to high-precision devices, the normal force has become one of the influential factors that cannot be ignored. The stability of the normal force in magnetorheological materials is even more critical to the performance and functionality of these devices. The stability of normal forces in magnetorheological materials is critical to the performance and functionality of these devices. Lower normal forces can reduce the friction and impact forces experienced by magnetorheological materials during motion or vibration processes, thereby minimizing mechanical vibration and noise generation and improving system stability. In addition, lower normal forces can reduce energy losses during strain processes in magnetorheological materials, thereby increasing system efficiency and extending device life. Therefore, there is an urgent need to investigate methods of controlling normal forces to improve stability.

Sepiolite minerals have desirable mechanical properties that make them suitable for applications requiring mechanical reinforcement and thermal insulation in a variety of industries [28,29,30]. According to research, sepiolite has been found to improve the settling stability and some of the rheological properties of magnetorheological lipids [31]. In this study, we prepared an aluminum–lithium-based magnetorheological grease mixture containing sepiolite, and the effects of magnetic field, time, shear rate, temperature, shear strain, and frequency on the normal force were analyzed separately for steady shear and oscillatory shear modes using a rotational rheometer (MCR-302). The normal force of this mixture exhibited a smaller and more stable range of variation compared to the original fluid under different magnetic field intensities and temperature conditions. The findings of this research provide some theoretical guidance for the design of high-precision devices and the understanding of material rheological performance.

## 2. Materials and Methods

### 2.1. Sample Preparation

#### 2.1.1. Raw Materials

The preparation materials for the test samples consisted of dimethyl silicone oil, carbonyl iron powder, 12-hydroxystearic acid, sebacic acid, lithium hydroxide monohydrate, diphenylamine, isopropyl aluminum alcoholate, benzoic acid, and mineral clay sepiolite. Of these, sepiolite was not included in the preparation of ALC. The carbonyl iron powder particles were products of the BASF company from Germany, with a size of 6 μm. Detailed information on the other materials is provided in Table 1.

#### 2.1.2. Principle of the Experiment

The preparation process can be divided into three parts:

(1) saponification reaction of 12-hydroxystearic acid and sebacic acid to generate lithium-based complex;
(2) reaction of isopropyl aluminum alcoholate, benzoic acid, and 12-hydroxystearic acid to form the aluminum-based complex;
(3) thickening of dimethyl silicone oil with the lithium-based complex and aluminum-based complex to form lithium–aluminum-based grease. Prior to thickening, carbonyl iron powder is added to produce ALC, and ALC is combined with sepiolite to form ALCH.

The chemical reaction equations are as follows.

(1) $${\mathrm{CH}}_{3}{{(\mathrm{CH}}_{2})}_{5}{\mathrm{CH}}_{2}{{(\mathrm{CH}}_{2})}_{10}\mathrm{COOH}+\mathrm{LiOH}\cdot H_{2}O\;\to {\;\mathrm{CH}}_{3}{{(\mathrm{CH}}_{2})}_{5}{\mathrm{CH}}_{2}{{(\mathrm{CH}}_{2})}_{10}{\mathrm{COOLi}+2H}_{2}O$$

(2) $${\mathrm{HOOC}(\mathrm{CH}}_{2}{)}_{8}\mathrm{COOH}+2\mathrm{LiOH}\cdot H_{2}O\;\to {\;\mathrm{LiOOC}(\mathrm{CH}}_{2}{)}_{8}{\mathrm{COOLi}+4H}_{2}O$$

(3) $$\begin{array}{l} {\mathrm{CH}}_{3}{\left({\mathrm{CH}}_{2}\right)}_{5}{\mathrm{CH}}_{2}{\left({\mathrm{CH}}_{2}\right)}_{10}{\mathrm{COOH}+H}_{2}{O+C}_{9}H_{21}{\mathrm{AlO}}_{3}+{\;C}_{6}H_{5}\mathrm{COOH}\;\to \\ {\left({\mathrm{CH}}_{3}\right)}_{2}{\mathrm{CHOH}+C}_{28}H_{47}{\mathrm{AlO}}_{5}\; \end{array}$$

**Figure 1:**
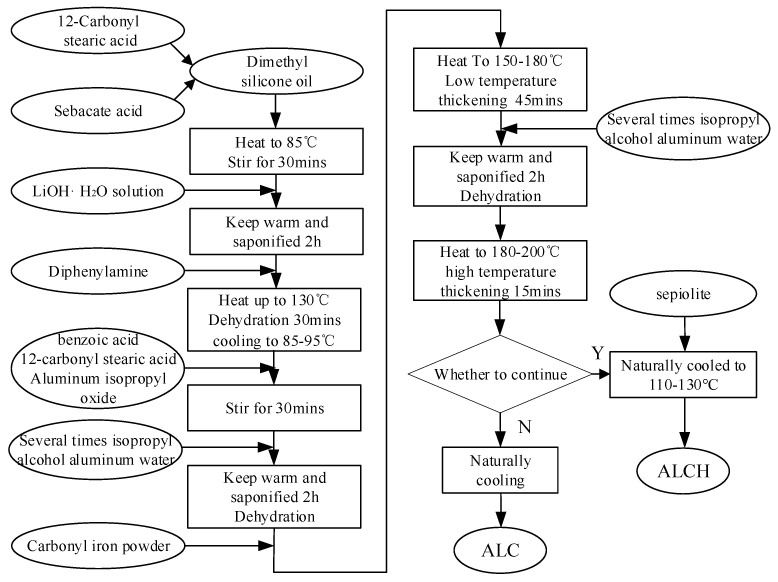
Sample preparation process.

The prepared aluminum–lithium-based magnetorheological grease contains a mass fraction of 70% of carbonyl iron powder, 0.75% of sepiolite, and 1.8% of thickening agent. The specific preparation process is shown in Figure 1.

### 2.2. Test Equipment and Experimental Principle

The experiment utilized a Physical MCR302 rheometer manufactured by Anton Paar from Austria to perform tests on the normal forces of two sample types in both steady shear and oscillatory shear modes. As depicted in the figure, the rheometer consists of a control module, a testing probe, a lower disk, and a magnetic field module. The control module is connected to a computer, and the lower disk is where the test sample is placed and allows for variations in the testing environment.

**Figure 2:**
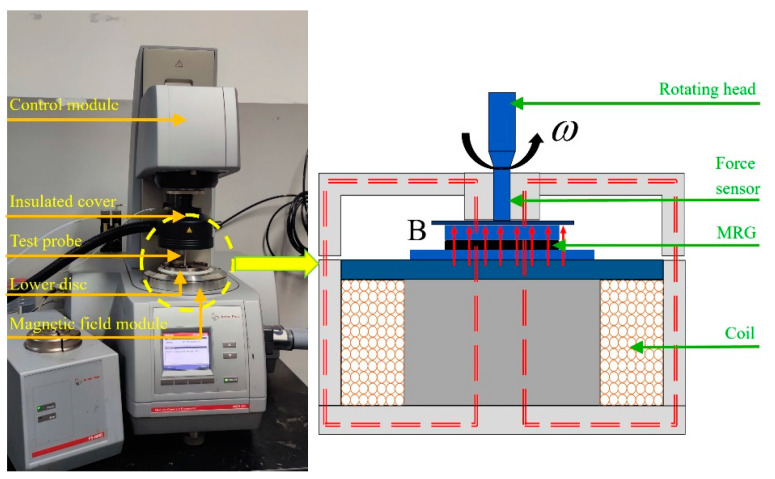
Normal force test equipment and principle.

The principle of testing normal forces is illustrated in Figure 2. When a magnetic field is applied, the magnetorheological grease inside the lower disk forms chains of magnetic particles, which exert a vertical force on the PP20 test head. This force is measured by sensors connected to the probe. By modifying parameters such as magnetic field strength, strain amplitude, and duration of action, the variation in normal forces under different conditions can be obtained.

## 3. Results and Analysis

### 3.1. Normal Force of ALC and ALCH in Steady-State Mode

#### 3.1.1. The Effect of Magnetic Fields on Normal Forces

**Figure 3:**
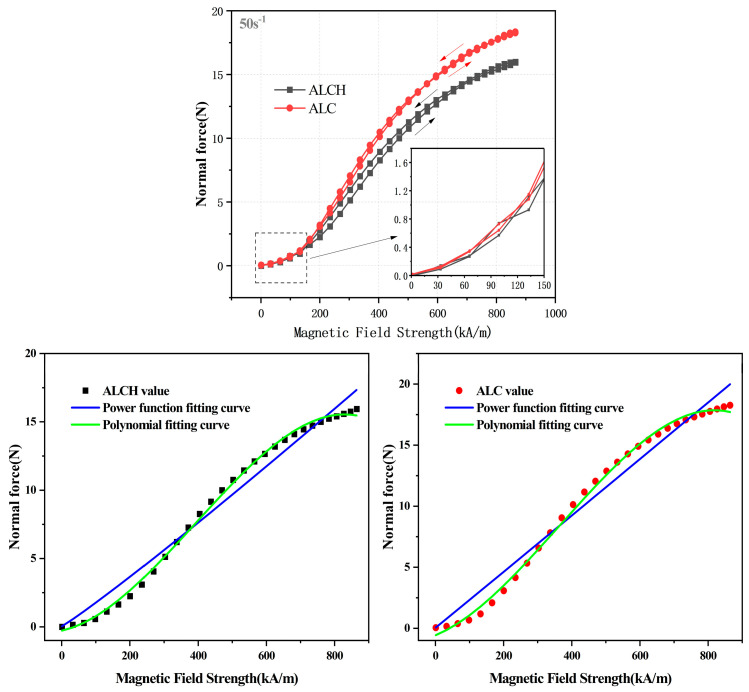
Relationship and fitting curve between the normal force and magnetic field of ALC and ALCH in steady-state mode.

To investigate the relationship between the normal force of the sample under steady shear and the magnetic field intensity, a shear rate of 50 s^−1^ was set, and the magnetic field intensity was scanned from 0 to 860 kA/m, and then reduced to 0 kA/m. The results are shown in Figure 3.

From the figure, in the steady state shear mode, it can be observed that the normal force increases as the magnetic field increases, and the normal force of ALC is always greater than that of ALCH. At 860 kA/m, the normal forces of ALC and ALCH are 18.6 N and 16.4 N, respectively. This is because the magnetorheological grease, which is subjected to laminar flow motion under the influence of shear motion, plays a destructive role in the chain structure, especially after the addition of sepiolite, and the more complex three-dimensional structure composed of sepiolite fibers and soap fibers exacerbates the destruction of the chain structure of the particles, which in turn leads to the normal force of ALCH being smaller than that of ALC. Furthermore, we can see that as the magnetic field scanning decreases, the normal force is greater than when it gradually increases. This is because under the influence of the magnetic field, the formation of particle chains also generates interparticle interaction forces, which hinder the generation of normal force. When the magnetic field increases, the interparticle interaction forces also increase [32]. When the magnetic field is unloaded, the decay rate of the interparticle interaction forces is faster than that of the normal force, resulting in a lagging trend of the normal force.

According to the research conducted by Liu et al. [33] on the fitting relationship between magnetic flux density and normal force, the relationship between magnetic field intensity and normal force is fitted, as shown in Equation (4):

(4) $$F=k\cdot H^{\alpha }$$

However, it was found that the fitting result had a large error. Therefore, Yao et al. [34] proposed a fitting research method using a third-degree polynomial for shear stress and magnetic field intensity, as shown in Equation (5):

(5) $$F=P1\cdot H^{3}+P2\cdot H^{2}+P3\cdot H^{1}+P4$$

The relationship between the normal force and magnetic field intensity was fitted using this method, yielding superior results. The fitting curve is shown in Figure 3 and fitting result parameters are presented in Table 2.

#### 3.1.2. The Effect of Time on Normal Forces

**Figure 4:**
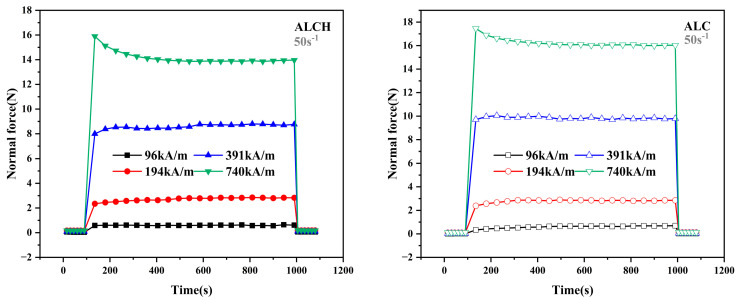
Relationship between the normal force and time of ALC and ALCH in steady-state mode.

Four different constant magnetic fields were set (96 kA/m, 194 kA/m, 391 kA/m, and 740 kA/m), along with a constant shear rate (50 s^−1^). After referring to the setup conditions of Ye [35], we collected a total of 32 data points in this test, in which the first six data points and the last six data points had no magnetic field and each data point was 15 s apart; the middle 20 test points had a magnetic field and each data point was 45 s apart, and the total test time was 1080 s. The aim was to investigate the variation of normal force over time for the two samples. The results are shown in Figure 4.

The figure shows that when no magnetic field is applied, the resulting normal force is negligible, but once the field is applied, the normal force increases significantly. It can be seen that the prolonged shear motion is also another factor that affects the normal force. At magnetic field strengths of 96 kA/m, 194 kA/m, and even 391 kA/m, the normal force remains relatively constant and is not affected by the shear time. However, the normal force shows a decreasing trend followed by a stable state when the magnetic field strength reaches 740 kA/m. This phenomenon can be attributed to the following reasons: at lower magnetic field strengths, the normal force generated is small, resulting in minimal shear friction between the plates, resulting in a relatively stable condition. On the other hand, at higher magnetic field strengths, the shear friction between the magnetorheological grease and the plates increases, causing damage to some chain-like structures. This damage gradually equilibrates with the magnetic field strength over time, leading to a state where the normal force first decreases and then stabilizes.

#### 3.1.3. The Effect of Temperature on Normal Forces

**Figure 5:**
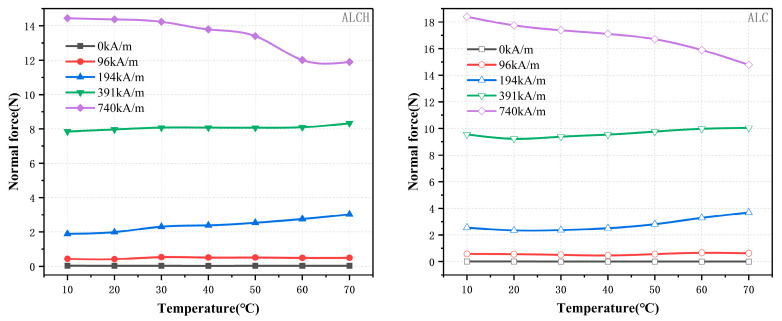
Relationship between the normal force and temperature of ALC and ALCH in steady-state mode.

Taking into account both environmental factors and heat produced during operation, temperature has a significant impact on the mechanical performance of magnetorheological fluids. The properties of the material and consequently the performance of the device as a whole are a function of temperature. It is therefore necessary to take into account the effect of the temperature on the performance of the normal force of the material under shear modes. The experiment involved varying the temperature between 10 and 70 °C, with a shear rate of 50 s^−1^ (Figure 5).

As shown in the figure, at magnetic field intensities of 0 kA/m and 96 kA/m, the normal force is almost independent of temperature. At magnetic field intensities of 194 kA/m and 391 kA/m, the normal force increases with temperature. The main cause of this phenomenon is the rise in temperature that results in a decrease in material viscosity and an increase in Brownian motion. Consequently, the chances of particle aggregation rise. This favors the formation and stabilization of the chain structure, which in turn leads to an increase in the normal force. With a magnetic field intensity of 740 kA/m, the normal force decreases as the temperature rises. This effect is the result of the complex interplay between temperature and magnetic field. In the presence of strong magnetic field constraints, the augmented Brownian motion has a harmful impact on the existing particle chains, thereby leading to a reduction in the normal force.

In summary, in a weak magnetic field, the normal force remains relatively unchanged and is minimally affected by temperature. In a moderate magnetic field, the normal force increases with temperature. In a strong magnetic field, there is a competitive relationship between the magnetic field and temperature, with the normal force decreasing as temperature rises. Sepiolite improves the stability of the normal force by 30% at a magnetic field strength of 740 kA/m.

#### 3.1.4. The Effect of Shear Rate on Normal Forces

**Figure 6:**
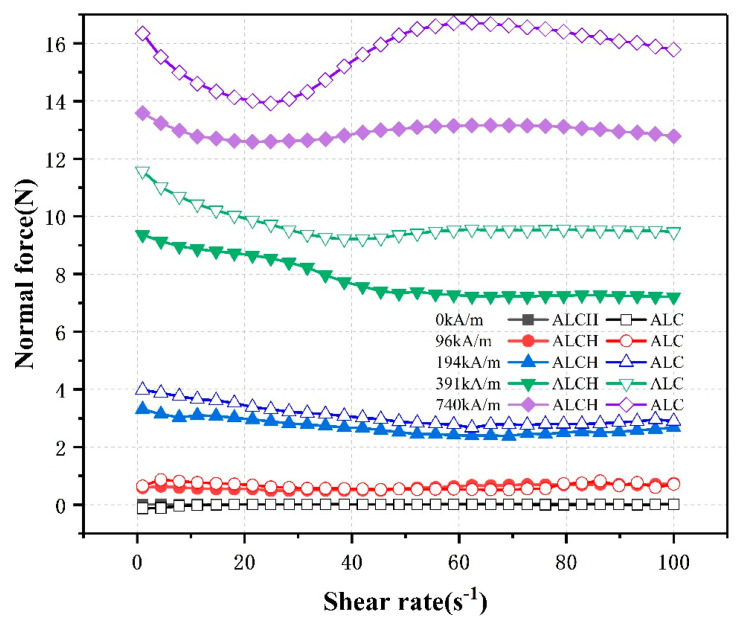
Relationship between the normal force and share rate of ALC and ALCH in steady-state mode.

As shown in Figure 6, the figure represents the relationship between shear rate and the normal force of the tested samples. The tested samples consist of two types: aluminum–lithium-based magnetorheological grease and aluminum–lithium-based magnetorheological grease containing sepiolite. Five different magnetic field intensities (0 kA/m, 96 kA/m, 194 kA/m, 391 kA/m, and 740 kA/m) were applied to these samples. They display the following features: In weak magnetic fields (0 kA/m and 96 kA/m), the normal force remains relatively constant, with little influence from the shear rate. In moderate magnetic fields (194 kA/m and 391 kA/m), the normal force gradually decreases with increasing shear rate (1 to 100 s^−1^).

This is because the increase in shear rate leads to the destruction of the chain structure of particles, causing a decrease in the normal force, the destruction of free particles will be generated in the magnetic field under the action of the new chain structure. Initially, when the shear rate is small, the free particles are fewer and the chance of recombination is small, the ability to rebuild the chain structure is weak, which is manifested as a decrease in the normal force. As the shear rate further increases, the number of particles increases and the chance of recombination increases, and the reconstruction ability is strengthened by the action of the magnetic field and gradually reaches equilibrium with the destruction, so that the normal force is maintained and tends to be stable, such as in the medium weak magnetic field, and the higher magnetic field strength further strengthens the chain structure, which is manifested as a decrease in the normal force. In the high magnetic field, the higher magnetic field strength will further strengthen the reconstruction of the chain structure, leading to the reconstruction and shear damage reaching a higher equilibrium point, resulting in a higher value of the normal force; thus, in the magnetic field of 740 kA/m, the normal force first decreases and then increases, and then tends to be stable. This phenomenon is then more obvious in the performance of the ALC, indicating that sepiolite is conducive to improving the stability of the normal force.

#### 3.1.5. Transient Response

**Figure 7:**
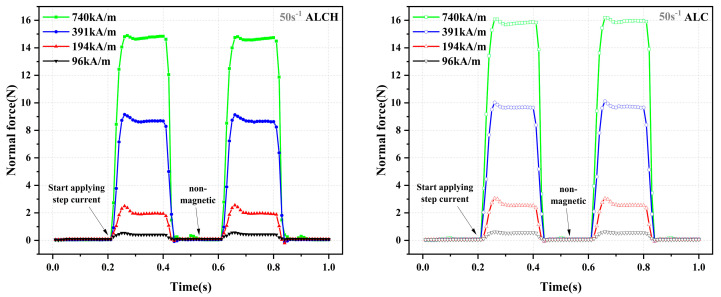
Transient response of ALC and ALCH under cyclic step field excitation.

Figure 7 illustrates the transient responses of ALCH and ALC under different step magnetic fields. It can be observed that the transient response time for both materials is within 70 ms. The fluctuations primarily correlate with the magnitude of the step magnetic field. The almost negligible difference in transient response time between ALC and ALCH indicates that sepiolite has minimal influence on the transient response of aluminum–lithium-based magnetorheological grease. This can be attributed to the fact that the transient response time of MR materials is primarily determined by the content and size of ferromagnetic particles, as well as the concentration of the base carrier fluid [9,10]. This can be compared with the study of Qian [31], which shows that the response times of normal force and shear stress are synchronized.

### 3.2. Normal Force of ALC and ALCH in Oscillation Mode

#### 3.2.1. The Effect of Magnetic Fields on Normal Forces

**Figure 8:**
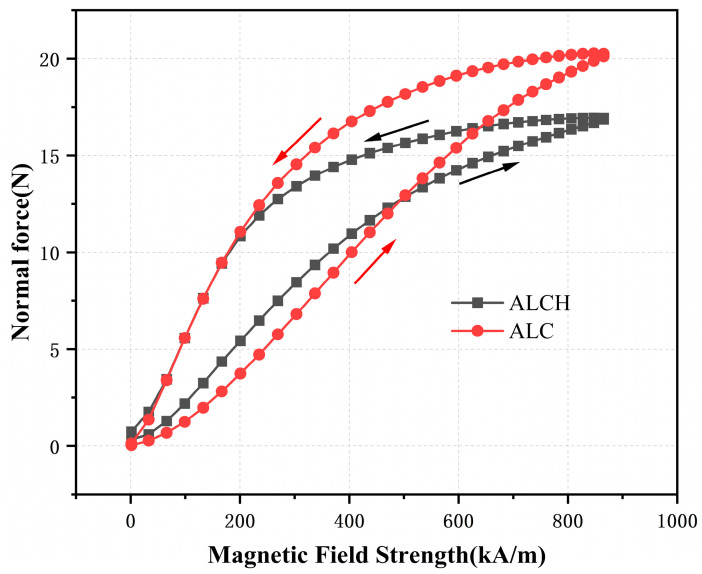
Relationship between the normal force and magnetic field of ALC and ALCH in oscillation mode.

Under the conditions of applying a strain excitation signal frequency f = 1 Hz and an amplitude r_0_ = 0.1%, magnetic field scans were performed on ALCH and ALC samples. The magnetic field intensity was scanned from 0 to 860 kA/m, and then from 860 back to 0 kA/m, resulting in the curve of the relationship between the normal force and the magnetic field intensity, as shown in Figure 8.

From Figure 8, it is evident that the normal forces of both samples increase with increasing magnetic field intensity. This suggests that in the presence of a magnetic field, magnetic particles continuously aggregate and form particle chain structures, resulting in a pushing on the equipment disc and the generation of normal forces. As the magnetic field intensity increases, the number of particle chains increases and becomes more stable, leading to subsequent increase in the normal forces.

It should be noted that the normal force of ALCH exceeds that of ALC when the magnetic field intensity is less than 500 kA/m during magnetic field loading. Nevertheless, the opposite is observed when the magnetic field intensity surpasses 500 kA/m. The primary cause of this phenomenon can be attributed to the fact that the saponified fiber structure prevails when the magnetic field intensity is less than 500 kA/m, and ALCH has a more complex fiber structure and therefore a higher normal force. Conversely, when the magnetic field intensity exceeds 500 kA/m, the higher magnetic field shifts the main contributor to the normal force from the fiber structure to the magnetic particle chain. At the same time, the complex fiber structure of ALCH affects the development of the chain structure, resulting in a lower normal force than that of ALC, which reverses the relative relationship and creates a discrepancy with the steady state shear results. Nevertheless, the absence of sepiolite in samples prompts more evident hysteresis phenomena.

#### 3.2.2. The Effect of Time on Normal Forces

The influence of the time history of the two samples on the normal force was investigated through experiments. The experiments were conducted under different magnetic field intensities (0 kA/m, 96 kA/m, 194 kA/m, 391 kA/m, 740 kA/m), with a frequency of 1 Hz and strain amplitude of 0.1%. The duration of the experiments was 250 s. The results obtained are shown in the figure below.

**Figure 9:**
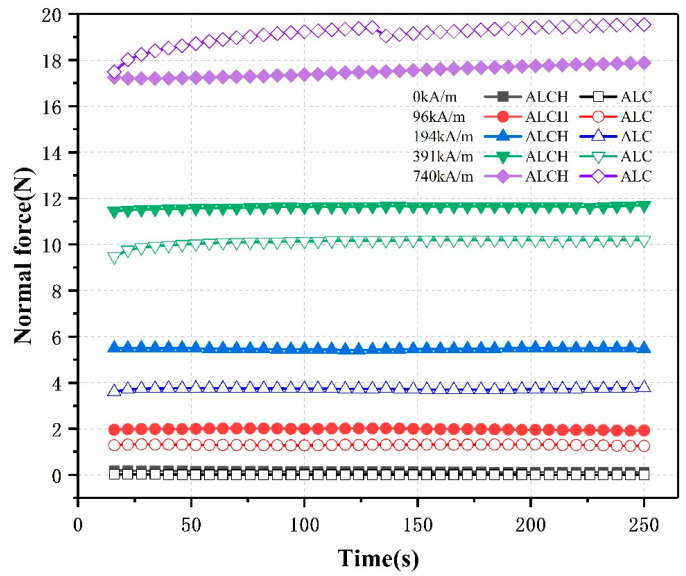
Relationship between the normal force and time of ALC and ALCH in oscillation mode.

Figure 9 represents the relationship between normal force and time for the two samples under different magnetic field intensities. It can be observed that the two samples exhibit similar trends in their variations at any constant magnetic field intensity. At weak and moderate magnetic fields (0 kA/m, 96 kA/m, 194 kA/m, and 391 kA/m), the normal force stabilizes at a constant level as the testing time increases. However, under the influence of a strong magnetic field (740 kA/m), the normal force increases with time. The phenomenon occurs due to the quick clustering of some magnetic particles into structures resembling clusters in a strong magnetic field. This leads to unequal magnetization of the magnetic particles in the sample and incomplete formation of particle chains. With time, the clusters gradually disappear, allowing complete formation of particle chains, resulting in a slight increase in the normal force. Thus, magnetic fluids necessitate more time in a strong magnetic field to gradually form a stable chain-like structure.

#### 3.2.3. The Effect of Temperature on Normal Forces

The relationship between temperature and normal force was examined for two samples. The temperature was varied within the range of 10 to 70 °C, while the magnetic field intensity was maintained at 96 kA/m, 194 kA/m,391 kA/m, and 740 kA/m. The resulting relationship curves are shown below.

**Figure 10:**
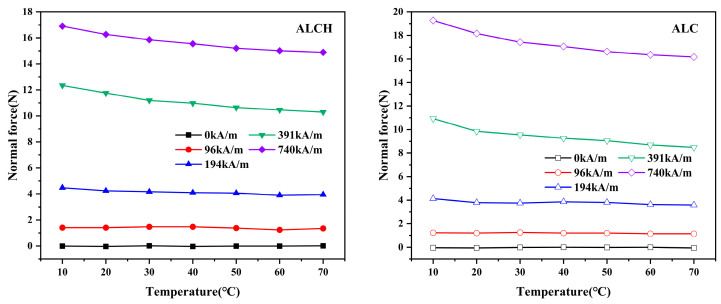
Relationship between the normal force and temperature of ALC and ALCH in oscillation mode.

The relationship between normal force and temperature for the two samples remains a consistent trend, as can be observed in Figure 10. At lower magnetic field strengths, the normal force demonstrates steady-state characteristics. However, in high-intensity magnetic fields, it decreases as the temperature increases. At lower magnetic field strengths, the particle structure is relatively loose and the Brownian motion induced by temperature has less effect on the interaction force between the particles, so the normal force is almost constant. At higher magnetic fields, the interaction between particles becomes tighter. Elevated temperatures disrupt the orderly arrangement of particles, leading to a decrease in the normal force with increasing temperature. Magnetorheological greases have different sensitivities to temperature at different magnetic field strengths, and magnetorheological greases with higher magnetic field strengths are more affected by temperature. It can be observed that the normal force of ALCH exhibits a smaller variation range with temperature when compared to ALC. For instance, when exposed to a magnetic field of 740 kA/m, the normal force of ALC drops from 19.27 to 16.16 N, and the normal force of ALCH decreases from 16.91 to 14.9 N, leading to a stability improvement of 36%. Therefore, sepiolite can reduce the temperature sensitivity of magnetorheological grease to some extent.

#### 3.2.4. The Effect of Frequency on Normal Forces

**Figure 11:**
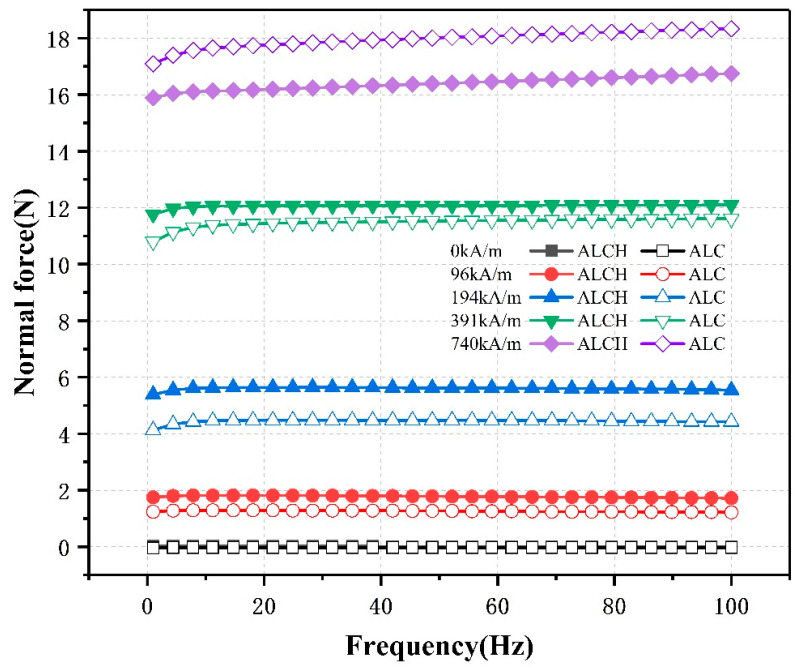
Relationship between the normal force and frequency of ALC and ALCH in oscillation mode.

In order to investigate the influence of frequency on the normal force of two samples under different constant magnetic fields, a shear strain amplitude (r_0_) of 0.01% was set. The magnetic field intensities were fixed at 0 kA/m, 96 kA/m, 194 kA/m, 391 kA/m, and 740 kA/m, respectively. Frequency scans were performed on both ALC and ALCH samples, with frequency ranging from 1 to 100 Hz. The measured results of the normal force are shown in Figure 11.

From Figure 11, it can be observed that the normal force remains relatively constant across the entire frequency range, with only a slight increase observed at a magnetic field intensity of 740 kA/m. This indicates that the influence of frequency on the normal force is minimal. The variation in the normal force is primarily correlated with changes in the magnetic field intensity. At a frequency of 100 Hz, both the ALCH and ALC samples experience an increase in the normal force from 0 N and −0.03 N at zero magnetic field to 16.75 N and 18.34 N, respectively, at a magnetic field intensity of 740 kA/m. Additionally, in moderate to weak magnetic fields, the normal force of the ALCH sample is greater than that of the ALC sample, while in strong magnetic fields, the normal force of the ALC sample is greater than that of the ALCH sample. This conclusion aligns with the findings presented in previous sections. It is worth noting that this research method is similar to that of Zhang [36], and the conclusions are consistent with those of Ye [37], but contrary to the findings of Gong et al. [38] regarding the oscillatory behavior of the normal force of magnetic rheological fluids with changing frequency. The normal force of the two samples does not vary with frequency. This feature broadens the range of applications for these devices.

#### 3.2.5. The Effect of Strain Amplitude on Normal Force

**Figure 12:**
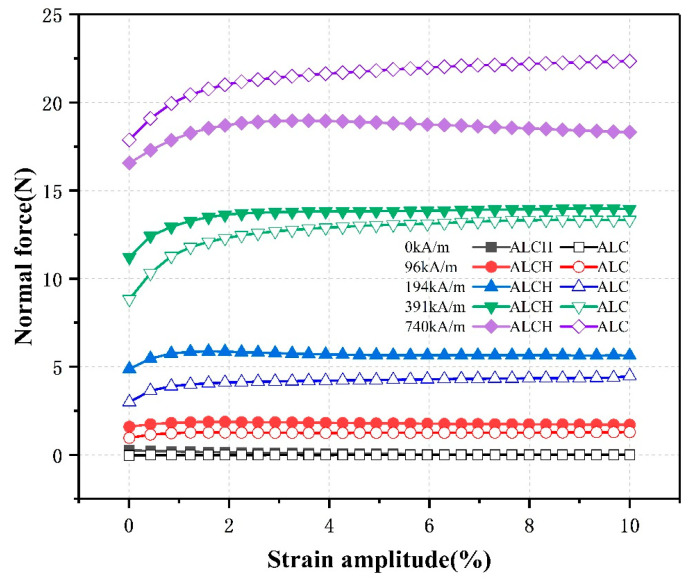
Relationship between the normal force and strain amplitude of ALC and ALCH in oscillation mode.

To investigate the relationship between strain amplitude and normal force, various constant magnetic field intensities (0 kA/m, 96 kA/m, 194 kA/m, 391 kA/m, and 740 kA/m) were established. The frequency was set at 1 Hz, and the strain amplitude was scanned from 0.1% to 10%. The normal force behavior of the ALCH and ALC samples was examined, and the results are depicted in Figure 12.

Figure 12 suggests that the relationship between the normal force and strain amplitude can be bifurcated into two parts. The normal force increases as the strain amplitude is increased when it is below the critical value; this effect is more distinct with a stronger magnetic field. This can be attributed to the driving force of the magnetic field causing numerous magnetic particles to aggregate and form a continuous chain-like structure. Similarly, when the strain amplitude is below the critical value, the chain-like configuration remains undisturbed and causes a steady increment in the initial normal force. The magnetic force determines the critical value [32]. Beyond the critical value, the acceleration of the normal force abates and shows a comparatively steady pattern. This is due to the incessant shear strain that keeps damaging the chain-like structure as the strain amplitude surpasses the critical value. The magnetic field, however, brings about a constant breakdown and reconstruction of particle chains causing them to attain fresh equilibrium leading to a steady normal force. Notably, the normal force of ALCH manifests a decline once the critical value is exceeded. This trend is accentuated particularly at a magnetic field intensity of 740 kA/m. The sepiolite fiber structure impedes the development of chain-like structures preventing it from reconstructing effectively as the destructive proportional effect of strain outweighs it. Thus, this leads to a drop in the normal force.

## 4. Conclusions

The present study investigates the normal force of self-made aluminum–lithium-based magnetorheological grease (ALC) and sepiolite-containing aluminum–lithium-based magnetorheological grease (ALCH) under steady shear and oscillatory shear modes. The measurements were performed considering various parameters, including magnetic field, time, strain amplitude, frequency, and temperature. The main findings are as follows:

(1) Under steady shear mode, ALC exhibits higher normal force than ALCH at the same magnetic field intensity. The range of normal force variations for ALCH is 10% smaller than for ALC at 860 kA/m. This is because after the addition of sepiolite, the more complex three-dimensional structure composed of sepiolite fibers and soap fibers exacerbates the destruction of the chain structure of the particles. Meanwhile the field-dependent normal force limitations of sepiolite on MRG are not disturbed by temperature, time, and shear rate. The normal force remains steady with time course in the medium–weak magnetic field, and remains steady after showing a decreasing trend at 740 kA/m; the shear rate leads to a decrease in the normal force, but different equilibriums are reached at different magnetic field strengths.
(2) Under oscillatory shear mode, ALC exhibits higher normal force than ALCH at high magnetic fields, but lower normal force at medium and weak magnetic fields. The range of normal force changes for ALCH is 30% less than for ALC. This is because at lower strain amplitudes, as the magnetic field increases, the main contributor of the normal force gradually changes from the fiber structure to the magnetic particle chain. The normal force is almost independent of time and frequency, but a critical value of the strain amplitude exists, which divides the change of normal force with amplitude into two regions: rising and stable.
(3) The effect of temperature on the normal force is mainly manifested under high magnetic field, and the increase in temperature leads to the decrease in normal force under high magnetic field, which is due to the fact that the particle chain is more constrained by the magnetic field under the high intensity magnetic field, and the Brownian motion caused by the increase in temperature will have a negative effect on the particle chain, causing the decrease in normal force. However, sepiolite improves the stability of the normal force in steady state and oscillatory modes by 30% and 36% respectively, indicating that sepiolite is able to reduce the sensitivity to temperature.

## Figures and Tables

**Table 1:** Reagent information.

Reagent Name	Specification	Manufacturer
Dimethyl silicone oil (kinematic viscosity 100 mm^2^/s)	PMX-200	Dow Corning Silicone Co., Ltd., Midland, MI, USA
Carbonyl iron powder	SQ	Provided by BASF in Ludwigshafen, Germany
12-hydroxystearic acid	H-00994	Tianjin Heowns Biochem Technologies, LLC., Tianjin, China
Sebacic acid	S-00200	Tianjin Heowns Biochem Technologies, LLC., Tianjin, China
Lithium hydroxide monohydrate	P02315	Shanghai Dingfen Chemical Technology Co., Ltd., Shanghai, China
Diphenylamine	M03782	Shanghai Myrell Chemical Technology Co., Ltd., Shanghai, China
Aluminum isopropyl alcohol	CP	Sinopharm Chemical Reagent Co., Ltd., Beijing, China
Benzoic acid	AR	Sinopharm Chemical Reagent Co., Ltd., Beijing, China
Sepiolite	Sep-01	Guzhang Shanlin Shiyu Mineral Products Co., Ltd., Changzhou, China

**Table 2:** The fitting parameter of samples at a shear rate of 50 s^−1^.

Sample	Parameter							
	k	α	R1	P1	P2	P3	P4	R2
**ALC**	0.02232	1.005	0.9722	−5.016 × 10^−8^	5.473 × 10^−5^	0.01136	−0.6095	0.99711
**ALCH**	0.01264	1.068	0.97556	−4.766 × 10^−8^	5.612 × 10^−5^	0.005356	−0.3147	0.99811

## Data Availability

Raw/processed data cannot be shared at this time, as the data is also part of ongoing research.
